# Supplementary material for: A Humanized Yeast Phenomic Model of Deoxycytidine Kinase to Predict Genetic Buffering of Nucleoside Analog Cytotoxicity
Source: Genes (Basel). 2019 Sep 30;10(10):770. doi: 10.3390/genes10100770 (PMC6826991; doi:10.3390/genes10100770)
Supplement: Supplementary file 1 [file genes-10-00770-s001.zip › Additional_Files/Additional_File7_GOTermHeatmaps.zip!/Additional_File7_GOTermHeatmaps/RNA_phosphodiester_bond_hydrolysis,_endonucleolytic.pdf]

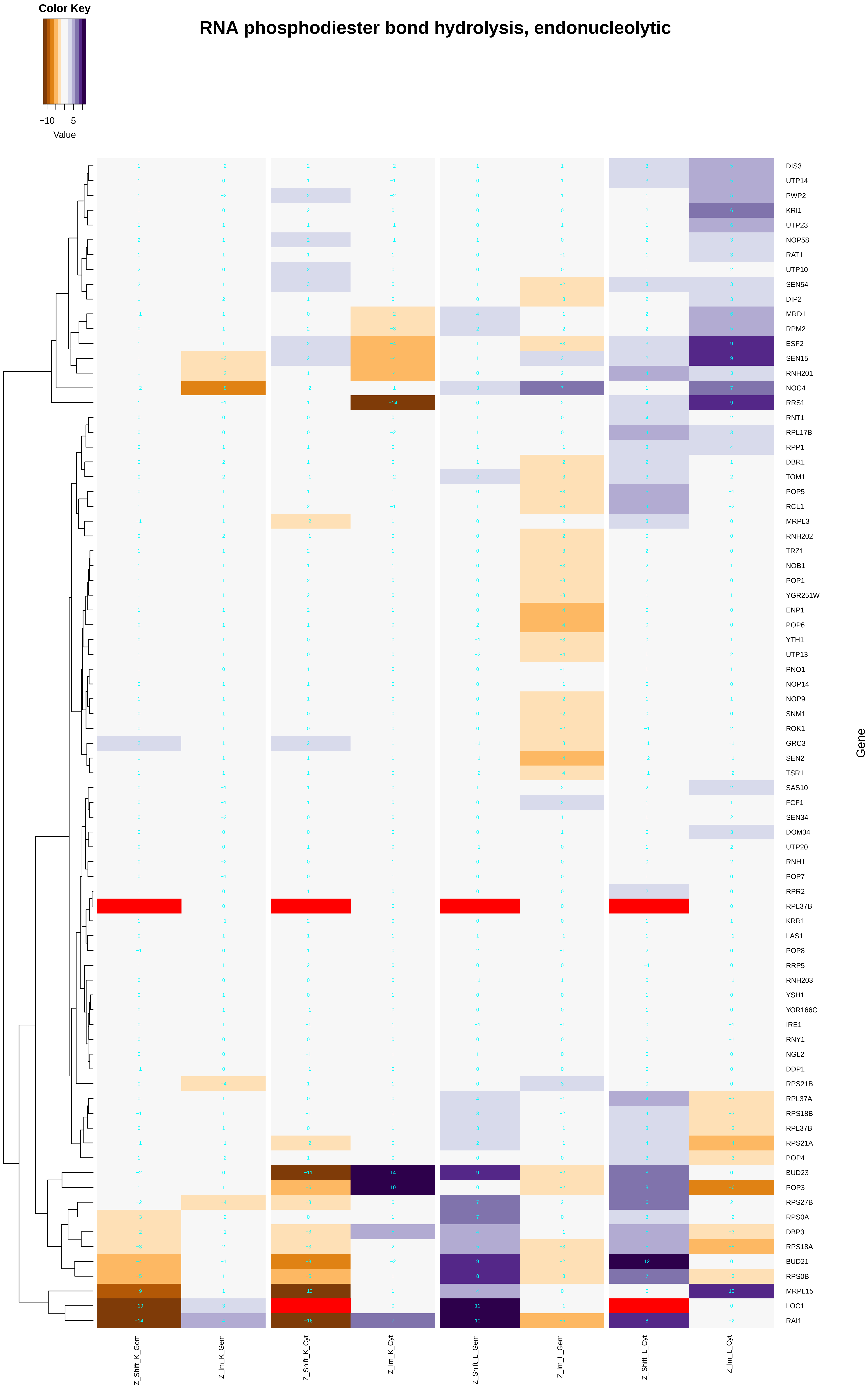

RNA phosphodiester bond hydrolysis, endonucleolytic
Color Key
−10
5
Value
Gene
DIS3
UTP14
PWP2
KRI1
UTP23
NOP58
RAT1
UTP10
SEN54
DIP2
MRD1
RPM2
ESF2
SEN15
RNH201
NOC4
RRS1
RNT1
RPL17B
RPP1
DBR1
TOM1
POP5
RCL1
MRPL3
RNH202
TRZ1
NOB1
POP1
YGR251W
ENP1
POP6
YTH1
UTP13
PNO1
NOP14
NOP9
SNM1
ROK1
GRC3
SEN2
TSR1
SAS10
FCF1
SEN34
DOM34
UTP20
RNH1
POP7
RPR2
RPL37B
KRR1
LAS1
POP8
RRP5
RNH203
YSH1
YOR166C
IRE1
RNY1
NGL2
DDP1
RPS21B
RPL37A
RPS18B
RPL37B
RPS21A
POP4
BUD23
POP3
RPS27B
RPS0A
DBP3
RPS18A
BUD21
RPS0B
MRPL15
LOC1
RAI1
Z_Shift_K_Gem
Z_Im_K_Gem
Z_Shift_K_Cyt
Z_Im_K_Cyt
Z_Shift_L_Gem
Z_Im_L_Gem
Z_Shift_L_Cyt
Z_Im_L_Cyt

endonucleolytic cleavage involved in rRNA processing

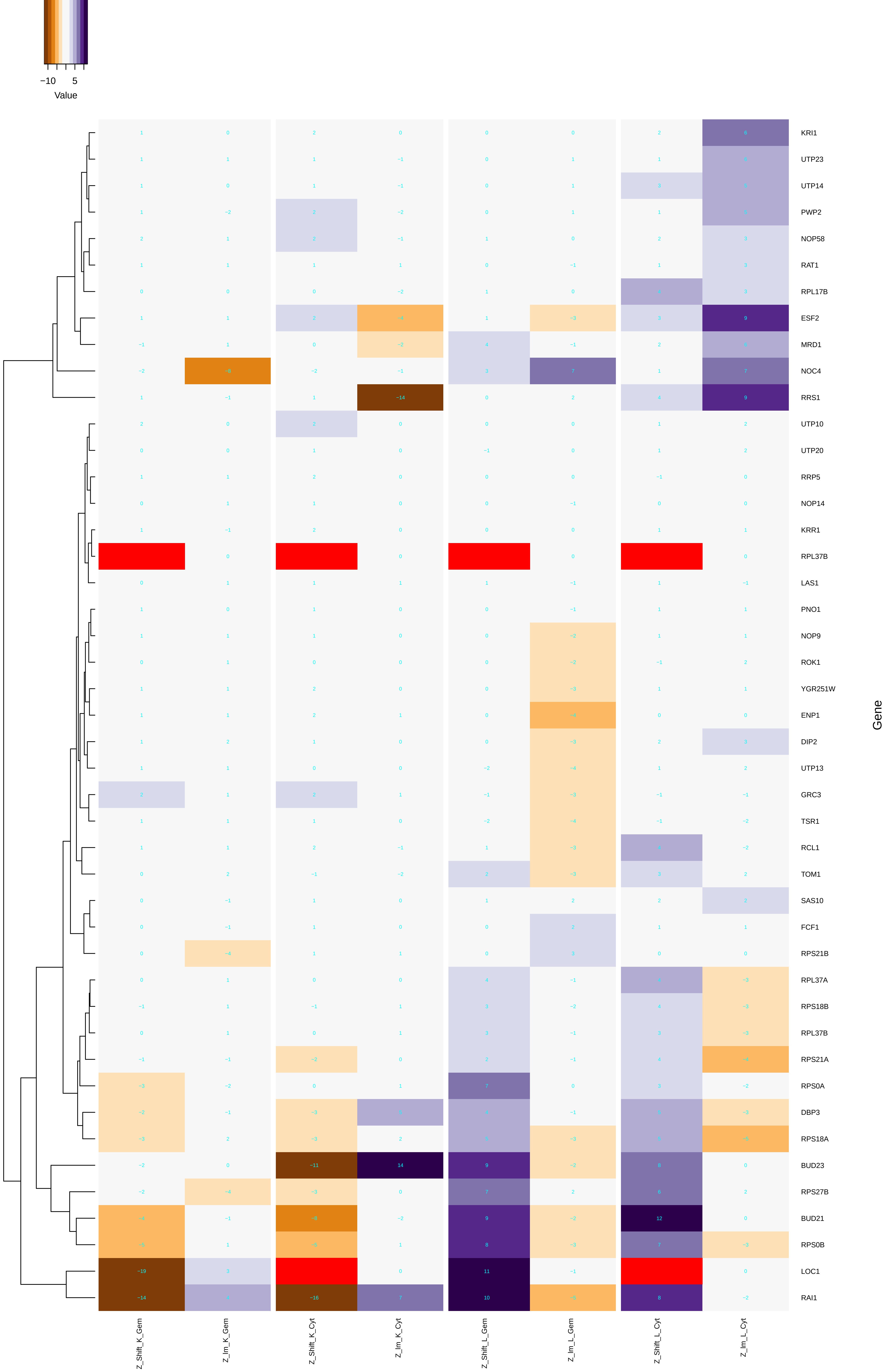

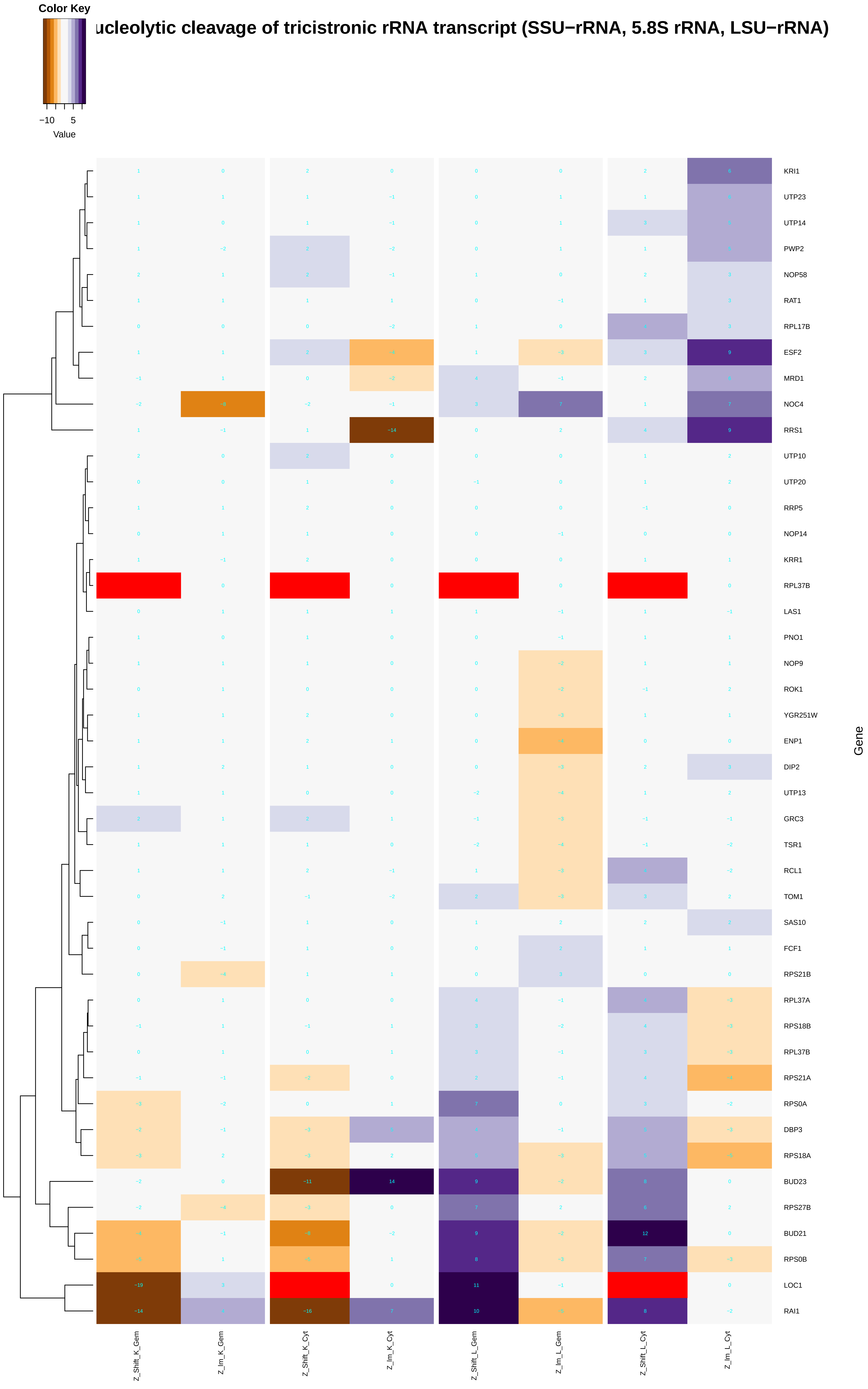
Color Key
−10
5
Value
ucleolytic cleavage of tricistronic rRNA transcript (SSU−rRNA, 5.8S rRNA, LSU−rRNA)
KRI1
UTP23
UTP14
PWP2
NOP58
RAT1
RPL17B
ESF2
MRD1
NOC4
RRS1
UTP10
UTP20
RRP5
NOP14
KRR1
RPL37B
LAS1
PNO1
NOP9
ROK1
YGR251W
ENP1
DIP2
UTP13
GRC3
TSR1
RCL1
TOM1
SAS10
FCF1
RPS21B
RPL37A
RPS18B
RPL37B
RPS21A
RPS0A
DBP3
RPS18A
BUD23
RPS27B
BUD21
RPS0B
LOC1
RAI1
Gene
Z_Shift_K_Gem
Z_Im_K_Gem
Z_Shift_K_Cyt
Z_Im_K_Cyt
Z_Shift_L_Gem
Z_Im_L_Gem
Z_Shift_L_Cyt
Z_Im_L_Cyt

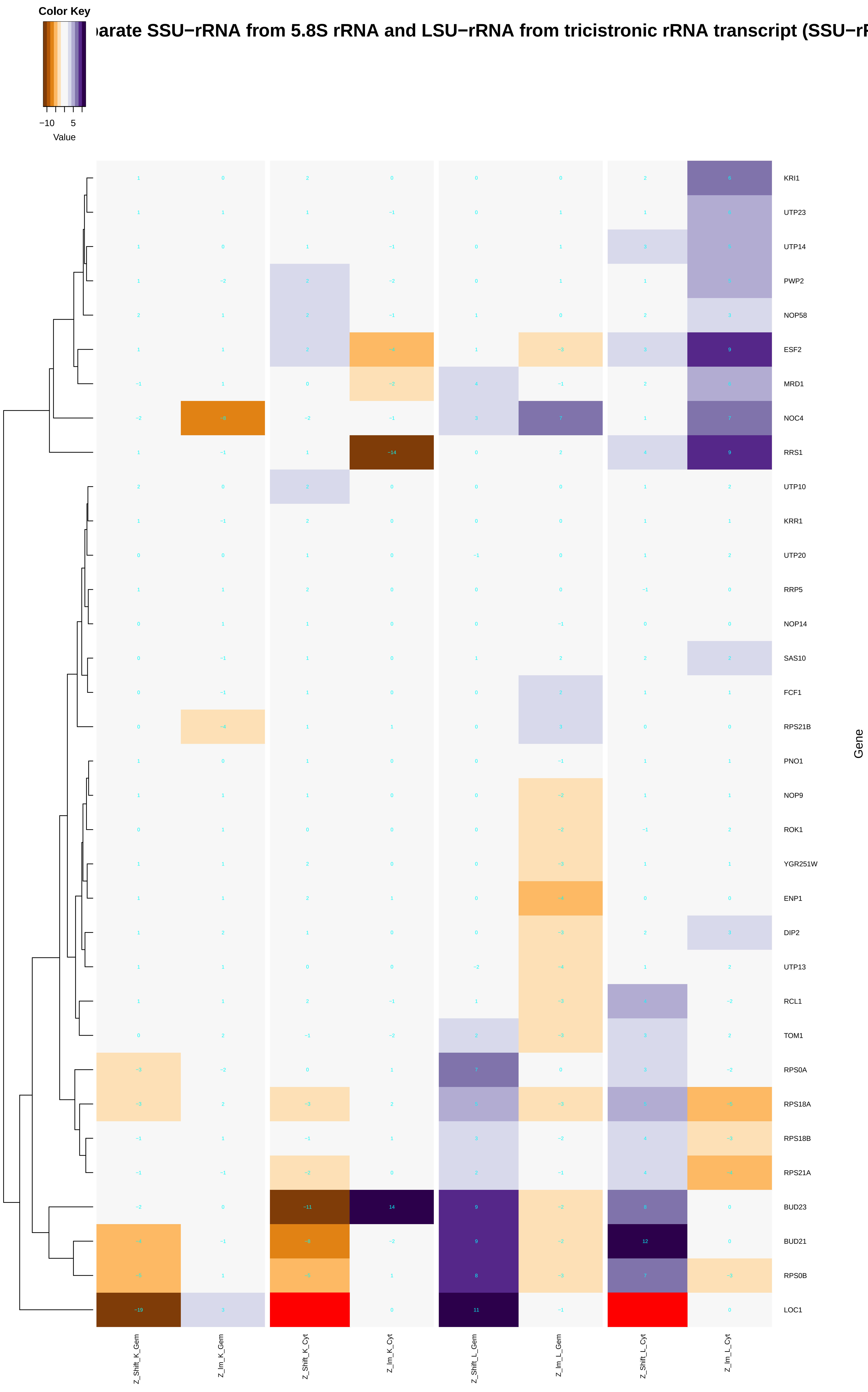
Color Key
−10 5
Value
arate SSU−rRNA from 5.8S rRNA and LSU−rRNA from tricistronic rRNA transcript (SSU−r
KRI1
UTP23
UTP14
PWP2
NOP58
ESF2
MRD1
NOC4
RRS1
UTP10
KRR1
UTP20
RRP5
NOP14
SAS10
FCF1
RPS21B
PNO1
NOP9
ROK1
YGR251W
ENP1
DIP2
UTP13
RCL1
TOM1
RPS0A
RPS18A
RPS18B
RPS21A
BUD23
BUD21
RPS0B
LOC1
Gene
Z_Shift_K_Gem
Z_Im_K_Gem
Z_Shift_K_Cyt
Z_Im_K_Cyt
Z_Shift_L_Gem
Z_Im_L_Gem
Z_Shift_L_Cyt
Z_Im_L_Cyt

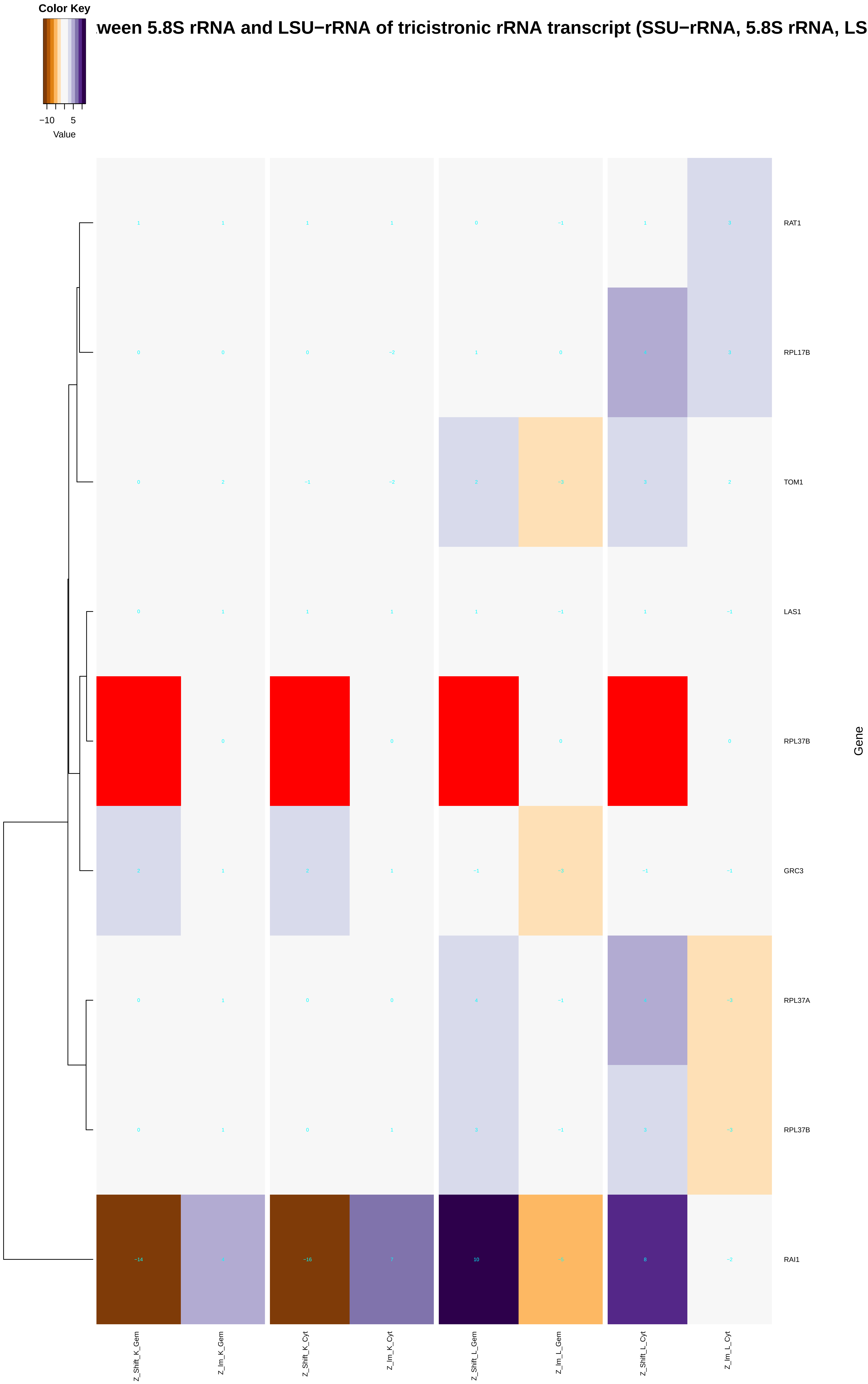
ween 5.8S rRNA and LSU−rRNA of tricistronic rRNA transcript (SSU−rRNA, 5.8S rRNA, LS
Color Key
−10
5
Value
RAT1
RPL17B
TOM1
LAS1
RPL37B
GRC3
RPL37A
RPL37B
RAI1
Gene
Z_Shift_K_Gem
Z_Im_K_Gem
Z_Shift_K_Cyt
Z_Im_K_Cyt
Z_Shift_L_Gem
Z_Im_L_Gem
Z_Shift_L_Cyt
Z_Im_L_Cyt

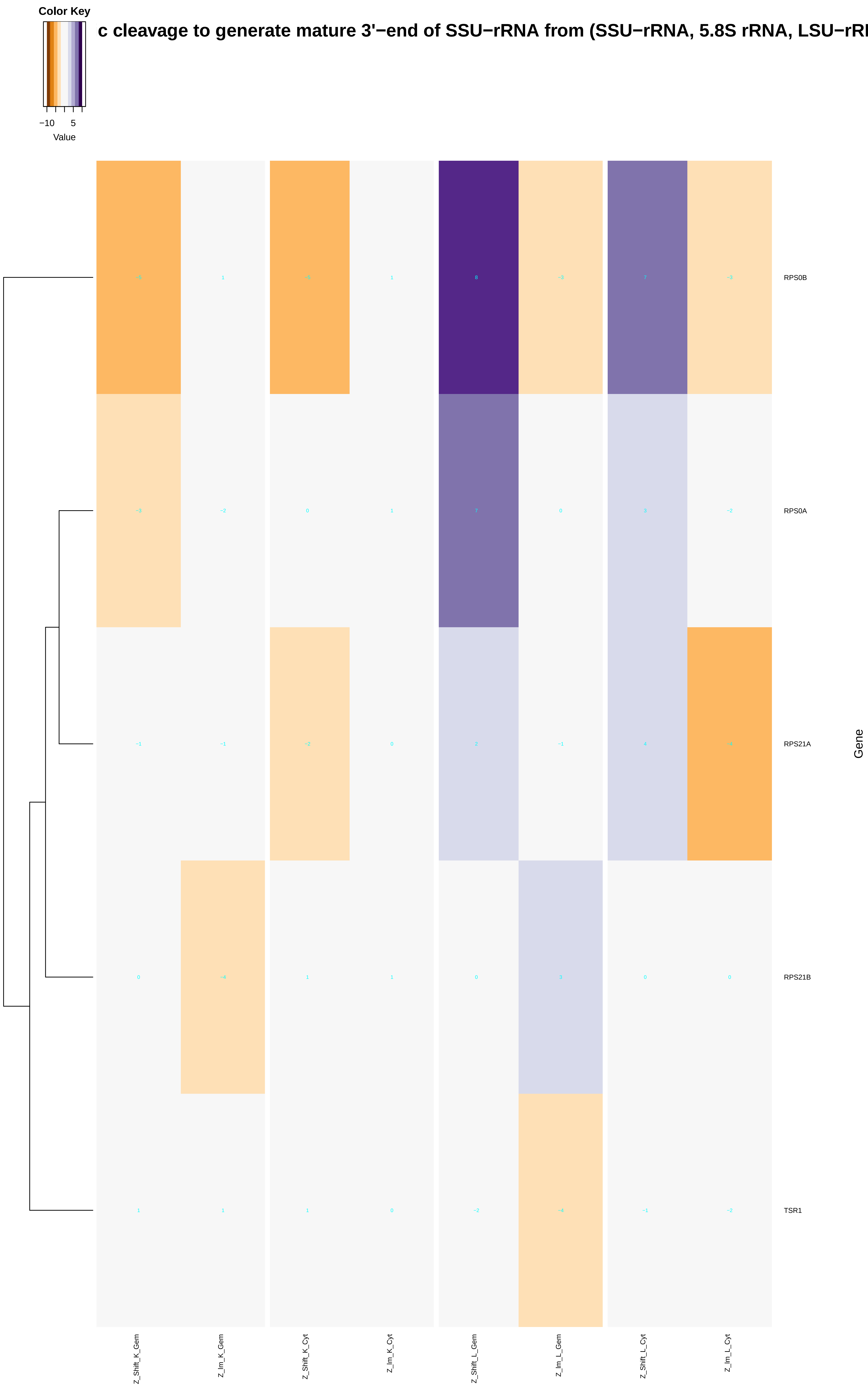
Color Key
−10
5
Value
c cleavage to generate mature 3'−end of SSU−rRNA from (SSU−rRNA, 5.8S rRNA, LSU−rR
RPS0B
RPS0A
RPS21A
RPS21B
TSR1
Gene
Z_Shift_K_Gem
Z_Im_K_Gem
Z_Shift_K_Cyt
Z_Im_K_Cyt
Z_Shift_L_Gem
Z_Im_L_Gem
Z_Shift_L_Cyt
Z_Im_L_Cyt

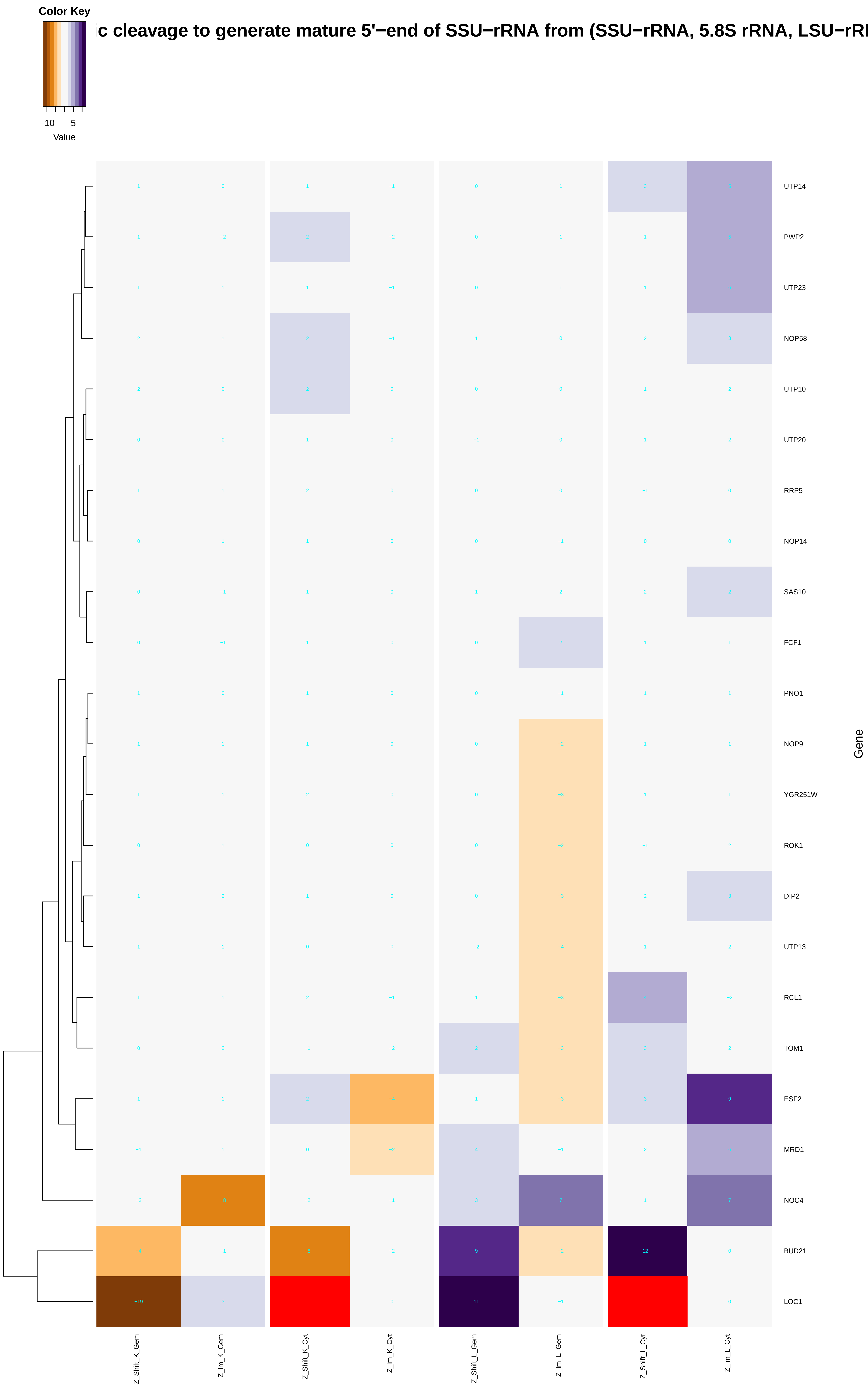

Color Key
−10
5
Value
c cleavage to generate mature 5'−end of SSU−rRNA from (SSU−rRNA, 5.8S rRNA, LSU−rRI
UTP14
PWP2
UTP23
NOP58
UTP10
UTP20
RRP5
NOP14
SAS10
FCF1
PNO1
NOP9
YGR251W
ROK1
DIP2
UTP13
RCL1
TOM1
ESF2
MRD1
NOC4
BUD21
LOC1
Gene
Z_Shift_K_Gem
Z_Im_K_Gem
Z_Shift_K_Cyt
Z_Im_K_Cyt
Z_Shift_L_Gem
Z_Im_L_Gem
Z_Shift_L_Cyt
Z_Im_L_Cyt

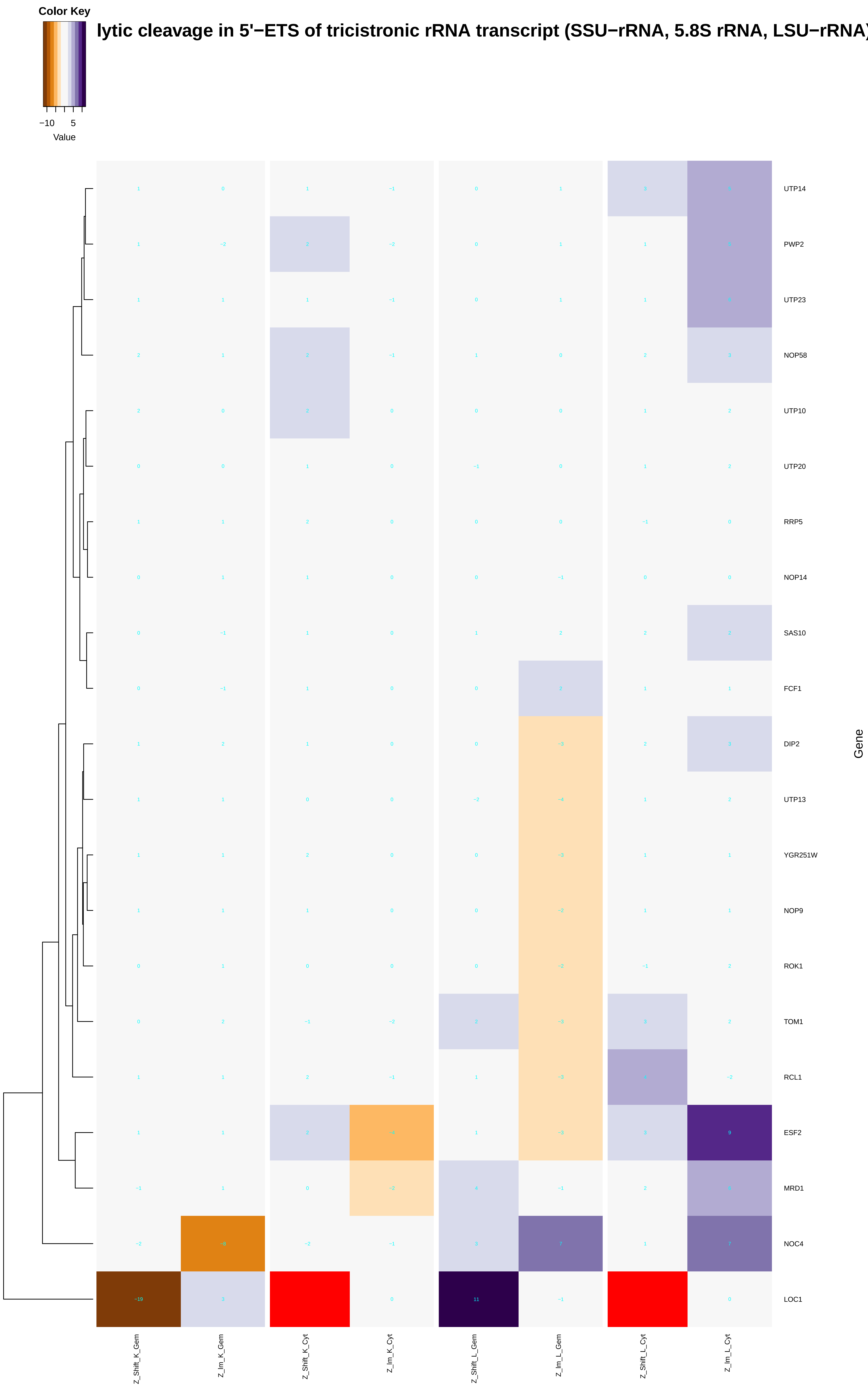
Color Key
lytic cleavage in 5'−ETS of tricistronic rRNA transcript (SSU−rRNA, 5.8S rRNA, LSU−rRNA)
−10
5
Value
UTP14
PWP2
UTP23
NOP58
UTP10
UTP20
RRP5
NOP14
SAS10
FCF1
DIP2
UTP13
YGR251W
NOP9
ROK1
TOM1
RCL1
ESF2
MRD1
NOC4
LOC1
Gene
Z_Shift_K_Gem
Z_lm_K_Gem
Z_Shift_K_Cyt
Z_lm_K_Cyt
Z_Shift_L_Gem
Z_lm_L_Gem
Z_Shift_L_Cyt
Z_lm_L_Cyt
